# Draft genome sequence of *Bradyrhizobium* sp. strain RDM4, a microsymbiont bacterium isolated from the root nodules of *Retama dasycarpa* in soils of Maâmora forest, Morocco

**DOI:** 10.1128/mra.00065-25

**Published:** 2025-05-07

**Authors:** Kaoutar Kaddouri, Mouad Lamrabet, Soufiane Alami, Zohra Chaddad, Hanaa Abdelmoumen, Bouabid Badaoui, Mustapha Missbah El Idrissi

**Affiliations:** 1 Center of Plant and Microbial Biotechnology, Biodiversity and Environment, Faculty of Sciences, Mohammed V University in Rabat 107736, Rabat, Morocco; The University of Arizona, Tucson, Arizona, USA

**Keywords:** *Retama dasycarpa*, *Bradyrhizobium*, draft genome, nanopore-sequencing, GridION

## Abstract

*Bradyrhizobium* sp. RDM4 is a symbiotic nitrogen-fixing bacterium, isolated from root nodules of the Moroccan endemic shrub *Retama dasycarpa* grown in Moroccan forest soils. In this work, we present the 8.4 Mb draft genome of this strain, characterized by a GC content of 63% and the presence of 8,141 total genes, with 7,032 protein-coding.

## ANNOUNCEMENT

Fabaceae plants play a crucial role in soil fertility, thanks to their aptitude to elicit symbiotic relationships with soil bacteria called rhizobia (1). *Retama dasycarpa* is a legume endemic to the High Atlas Mountains in Morocco (2). This shrub is well-adapted to extreme environments and contributes to the restoration of degraded ecosystems (3).

*Bradyrhizobium* sp. strain RDM4 was isolated from the root nodule of *R. dasycarpa* grown in Maâmora forest soils, Morocco (34°0.02 42 510 N; −6°. 72 38 750 W) (2). The nodules were surface disinfected with 0.1% mercury-chloride solution for 1 min and then crushed in 50 µL of sterile distilled water. The suspension obtained was streaked on Yeast-Extract-Mannitol (YEM) (4) agar medium at 28°C for 7 to 15 days. The obtained colonies were picked and re-streaked several times on solid YEM media until their purity and preserved at 4°C.

Genomic DNA was extracted from a fresh bacterial culture grown in 5 mL of liquid TY medium (5) for 5 days at 28°C, using the PureLink Genomic DNA MiniKit (Invitrogen) and quantified using a Thermo-Scientific NanoDrop 2000 spectrophotometer. The culture was prepared from one pure colony picked from the solid YEM medium.

The library was prepared using 400 ng of high-molecular-weight DNA, following the manufacturer’s recommendations for the Sequencing gDNA Barcoding Kit (SQK-RBK004). DNA sequencing was performed on a GridION device with a FLO-MIN106D, R.9.4.1 flow cell, according to the manufacturer’s instructions, for 72 hours (Oxford Nanopore Technologies, UK). Basecalling was conducted using the High Accuracy (HAC) model in Dorado software (v7.3.11), integrated within MinKNOW (v5.9.18).

The raw reads were quality-checked using FastQC version 1.2.1 (6). Adapter trimming was performed with Porechop version 0.2.4 (7), followed by filtering with NanoFilt version 2.8.0 (8). *De novo* genome assembly was conducted using Canu version 2.2 (9), polished four times with Racon version 1.5.0 (10), and once with Medaka version 2.0.1 (11). The sequencing generated 16,642 reads, with an N_50_ value of 16,731 bp (Table 1). Assembly quality was evaluated using Quast version 5.2.0 (12). Genome annotation was performed using the NCBI Prokaryotic Genome Annotation Pipeline. The closest type strain to the sequenced genome was identified using the Type Strain Genome Server (13). Digital DNA-DNA hybridization (dDDH) was calculated with the Genome-to-Genome Distance Calculator (GGDC v. 3.0) using formula 4 (14). The average nucleotide identity (ANI) was determined with pyani version 0.3.0-alpha (15). Default parameters were used for all software unless otherwise specified.

**TABLE 1:** Summary of sequencing, *de novo* assembly, and annotation results of the *Bradyrhizobium* sp. RDM4 genome

Strain characteristic(s)	*Bradyrhizobium* sp. RDM4
Sampling site	Soil of Maâmora forest Morocco
Host plant	*Retama dasycarpa*
No. of raw reads	22,510
No. of reads	16,642
No. of contigs	2
Mean read length (bp)	10,186,699
N50 (bp)	16,731
Genome length (bp)	8,405,022
Coverage (×)	20
N50 (bp)	8,193,764
GC content (%)	63%
Total genes	8,141
CDSs (coding DNA sequences)	7,032
No. of rRNA	3
No. of tRNAs	50
No. of tmRNAs	2
BioSample ID	SAMN44624647
BioProject ID	PRJNA1183372
NCBI RefSeq assembly	GCF_046197085.1

**Fig 1:**
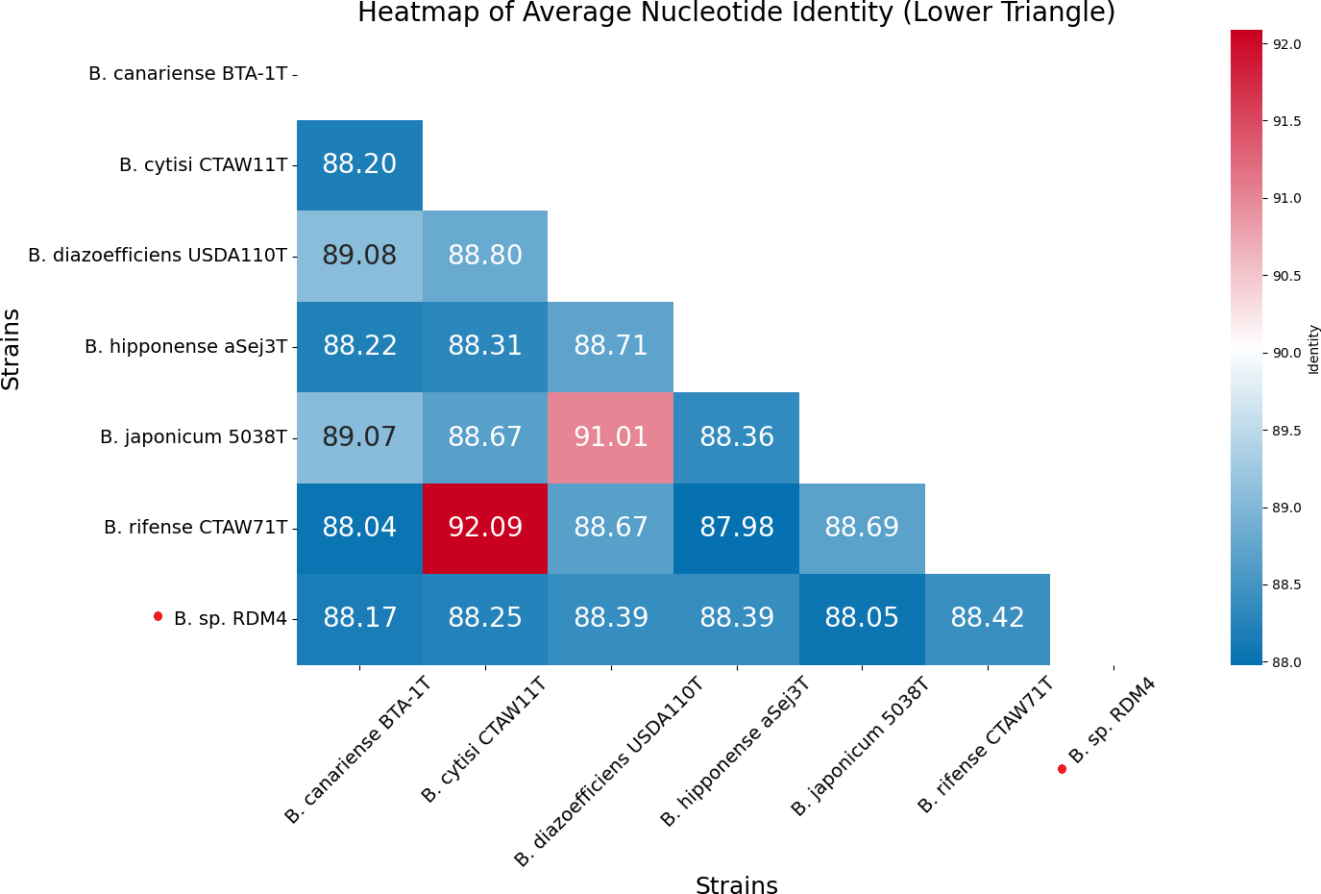
Heatmap of average nucleotide identity (ANI) percentages of *Bradyrhizobium* sp. RDM4 and closest-related type strains.

*De novo* assembly generated two contigs totaling 8,405,022 bp, with a GC content of 63% (Table 1). The first contig represents a circular nuclear genome of 8,193,764 bp with a GC content of 63%, while the second contig is a circular plasmid of 214,646 bp with a GC content of 60.5%. *Bradyrhizobium* sp. RDM4 was closely related to *B. rifense* CTAW71^T^, showing an ANI value of 88.42% and a dDDH of 33.5% (Fig. 1). The annotation identified a total of 7,032 protein-coding genes, 3 rRNA operons, 50 tRNAs, and 2 tmRNAs.

## Data Availability

The assemblies and sequence data of *Bradyrhizobium* sp. RDM4 are available in the NCBI database under accession number GCA_046197085.1, with BioProject number PRJNA1183372 and BioSample number SAMN44624647. The raw sequencing data can be accessed in the Sequence Read Archive (SRA) under accession number SRR31307243.
